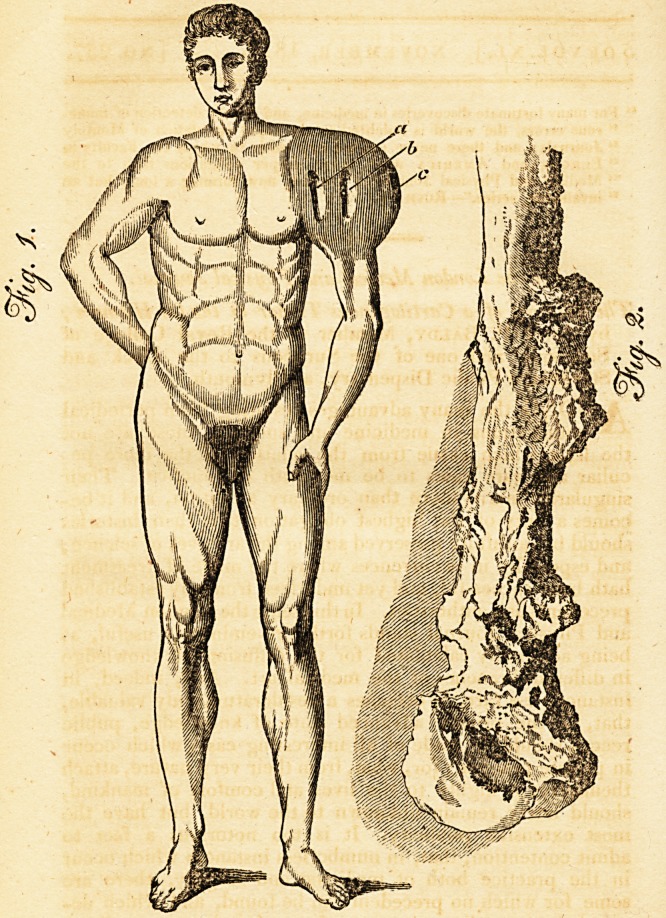# The History of a Cartilaginous Tumor of the Os Humeri

**Published:** 1818-11

**Authors:** John P. Baldy

**Affiliations:** Member of the Royal College of Surgeons, and one of the Surgeons to the Dock and Storehouse Public Dispensary, at Plymouth.


THE LONDON

### Medical and Physical Journal.

5 OF VOL. XL.] NOVEMBER, 1818. [NO. 237.

"For many fortunate discoveries in medicine, and for the detection of numerous errors, the world is indebted to the rapid circulation of Monthly "Journals; and there never existed any work to which the Faculty in "EUROPE and AMERICA were under deeper obligations than to the "Medical and Physical Journal of London, now forming a long, but an "invaluable, series."—RUSH.

*For the London Medical and Physical Journal.*

### *The History of a Cartilaginous Tumor of the Os Humeri;* by JOHN P. BALDY, Member of the Royal College of Surgeons, and one of the Surgeons to the Dock and Storehouse Public Dispensary, at Plymouth.

AMONG the many advantages resulting from periodical publications in medicine and surgery, those are not the least which ensue from the relation of the more peculiar and rare cases to be met with in practice. Their singularity merits more than ordinary attention, and it becomes a duty of the highest obligation that their histories should be faithfully preserved among the archives of science; and especially in occurrences where the mode of treatment hath been successful, and yet underived from any established precedents and authorities. In this sense the London Medical and Physical Journal stands forth pre-eminently useful, as being admirably calculated for the diffusion of knowledge in different branches of the medical art. And, indeed, in instances less rare, it becomes a desideratum truly valuable, that, in the present advanced state of knowledge, public record should be made of all interesting cases which occur in private practice; for, what, from their very nature, attach themselves so highly to the lives and comfort of mankind, should never remain unknown to the world, but have the most extensive publicity. It is too notorious a fact to admit contention, that, in numberless instances which occur in the practice both of medicine and surgery, there are some for which no precedent can be found, and which demand the immediate exertion of sound judgment to direct the means for their relief. And, on the other hand, even where long established opinions have given sanction to a mode of treatment which subsequent improvements have rendered questionable, it is ever to be lamented that any bias should be given to the mind from precedents unsupported by matter of fact. For it is more than possible the precedents, if analyzed, would be found dissimilar in many material points, and thereby often proving injurious, and not unfrequently fatal.

It is said of the great Lord Bacon, that, on his entrance on that sphere of action to which his labours were chiefly directed, and to the summit of which he at length attained, and was crowned with the most brilliant honours, he had to contend with men grown old in opinion, and whose pertinacity never gave way until fairly driven from their strong holds by overwhelming arguments, carrying every thing of ignorance before them. And if this great man, after thirty years theoretical and practical knowledge in the science to which his life was devoted, styled himself, on that account, as he did, the servant of posterity, how infinitely more devoted should be the lives of those who walk within the sacred inclosure of that science, where not the property, but the lives of men, are every day committed to their charge.

The interesting case, which I have the honour to transmit to the diplomatic body of practitioners, through the medium of your useful publication, is not perhaps wholly singular, but yet attended with circumstances certainly rare; and, from the eventual happy result with which it terminated, will be considered, I hope, worthy preservation among the records of your repository. And, as I venture to conclude, the more particular I am in the relation, the more earnest will be the attention of the profession to the case, I shall relate the progress of the disease from its commencement, through its different stages.

On the 11th of April, 1818, John Tonkin, æt. 23 years, made application for relief at the Dock and Storehouse Public Dispensary, on account of a considerable enlargement of the left shoulder. On enquiring into the history of the case, the poor man, in explaining the probable cause of his complaint, conceived that he had exerted himself one day in his labour beyond his strength, and thereby had induced some injury to his shoulder. About two or three days after this, perceiving his shoulder begin to swell, he made application to the medical man of the gun-wharf, where he then laboured; who, conceiving it to be rheumatism, treated it accordingly. The swelling continued increasing in size, attended with pain, for about four months; when one day, having a severe rigor, which brought on syncope, the same medical gentleman, who had before prescribed for him under the idea of its being rheumatism, was again requested to see him. Cataplasms were now ordered; and, after continuing them for some time, which tended not in the least degree to meliorate the disease, he ceased their application. It happened, much about this time, that a reduction took place of the labourers in the gun-wharf, and he was discharged. It was now, for the first time, that he made application at the Dock and Storehouse Public Dispensary, when he came under my care.

On examination of the joint, I discovered a swelling of considerable magnitude, extending over the whole of the deltoid muscle into the axilla, measuring about seventeen inches in circumference, with a partial discoloration of the integuments, preternatural heat, and an apparent fluctuation to be distinguished at that part of the swelling nearest the chest.

April 13th.—I called a consultation of my colleagues at the dispensary; the result of which was, that particular attention should be paid as to the progress of the tumour, and we should meet again on the 15th. I would here remark, that I judged it proper to give my patient a tonic draught at night, in order to check profuse perspirations which came on at this time.

April 15th.—The consultation was held this day; and it was the unanimous opinion that the swelling contained a fluid, though most probably deeply seated. It was thought expedient that an incision should be made into it at its most prominent part, to evacuate the fluid, that the nature of the disease might be more clearly ascertained. I accordingly made an incision through the centre of the tumour, carrying my knife down to the bone. In doing this, a small quantity of grumous fluid escaped; but what I wish more particularly to remark in this operation is, that my knife gave me the sensation as if I was cutting through a cartilaginous substance. The diseased mass exhibited an ash-coloured appearance, interspersed with dark livid spots.—Having satisfied ourselves in some measure as to the nature of the disease, the wound was dressed with adhesive plaster, and the patient put to bed. At my evening visit, he expressed himself very comfortable and easy.

April 16th.—He did not sleep during the night; had a considerable degree of fever, attended with great pain in the wound. I ordered him Ext. colocynth. c. gr. xij. Hyd. submur. gr. iij. ft. pil. iij. stat. sumend. I visited him again in the evening, when he expressed himself as having great pain in the wound; in consequence of which, I loosened the bandages a little.

April 17th.—Fever little abated; examined the wound; found it much inflamed and very tense, with the edges quite separated. Dressed it with the ung. cetac. We held another consultation this day; the result of which was, that nothing but amputation of the limb was likely to save his life; but that it was necessary to wait until the fever was lessened, and that Dr. Magrath (one of the physicians of the Dispensary) should be requested to superintend the medical treatment.

On the 1st of May, Dr. M. and myself called another consultation, the febrile symptoms having abated. The result was, that two large issues should be made, one at the anterior and another at the posterior part of the tumour; which was accordingly done.

I allowed the original wound and issues to discharge for the space of three weeks, when, instead of finding the swelling diminished, it was considerably augmented; so that, from the time of its first being measured to the present, it had increased full five inches.

May 21st.—Another consultation was held this day; the result of which was, that amputation of the limb should be immediately performed. The man's consent being at this time obtained, the following day was fixed for the purpose. —I would here observe, that the preternatural enlargement of the shoulder, and the diseased state of the integuments, together with the issues, precluded the possibility of attempting the operation in the usual manner. I was obliged, therefore, to pay particular attention to the morbid structure of the parts, in order to save as much integuments as possible, to cover the stump; in doing which, some difficulty was experienced.

22nd.—Every arrangement being made for the operation, it was performed in the following manner:—Having seated my patient in a chair, one of my colleagues compressed the subclavian artery above the clavicle, with a boot-hook well padded with cloth for that purpose. I made an incision through the integuments only, commencing from the upper and inner part of the shoulder down to the insertion of the deltoid muscle, and another incision from the upper and posterior part of the shoulder down to meet with the other in a point. The integuments were then dissected from the tumour, and turned back over the acromion scapulæ. An incision was then made at the upper and lateral parts of the joint, in order to cut the capsular ligament; which being accomplished, and the head of the bone detached from the glenoid cavity, I carried my knife down behind close to the bone, and cut through the muscles at that place just opposite the deltoid's insertion; by which the axillary artery was divided, and immediately secured. The limb being removed, a large portion of diseased organized substance still remained in the axilla, which I was obliged to dissect away in a very cautious manner, as it was imbedded in the plexus of nerves, and the axillary artery passing throught its very substance. Only two small vessels required to be tied; and, as the patient became now very much exhausted and faint, (not from the loss of blood, but from fatigue and pain,) he was immediately put to bed without the wound being dressed. Warm applications were used to the abdomen, feet, &c. and cordials were freely administered. These were continued for about half an hour, when he began to recover, and, as hæmorrhage did not follow, I proceeded to dress the wound. The portion of integuments that was dissected back was brought down, in form not unlike a wedge, between the two sides of the wound. Three sutures were applied, and the rest dressed with adhesive plaster.

At my evening visit, I found my patient very comfortable; there being no hæmorrhage, and very little pain.

23d.—Had passed a quiet night; very little fever, but great anxiety. Saw him again in the evening: fever much increased, attended with somewhat of delirium. I ordered Magnes. sulph. ℥i. Infus. sennæ, ℥ij. stat. sumend.

24th.—Fever much abated, delirium gone; the medicine had operated briskly.

25th.—Fever gone; dressed the wound, which assumed a very favourable appearance, a great part having healed by the first intention, and one of the ligatures fallen off.

26th.—Dressed him as before; ordered him to eat meat, and drink some wine in the course of the day.

As it would be tedious and uninteresting to relate the daily progress of the case, suffice it to say that the patient went on improving in health, and the wound healing, for the space of three weeks, when I discharged him cured.

On dissecting the arm after its removal, I was much surprised to find not the least appearance of any cellular or muscular fibre, but only one solid mass of diseased organized substance. The bone, as may be seen in the Plate, fig. 2. was very much diseased, having several large exostoses arising from it.

*Explanation of the Plate.*—Fig. 1. *a*, the anterior issue; *c*, the posterior; *b* points out the situation of the wound made previous to the amputation of the limb.

*Plymouth Dock; Oct.* 1, 1818.

## Figures and Tables

**Fig. 1. Fig. 2. f1:**